# Cultural Phenomena Believed to Be Associated With Orthorexia Nervosa – Opinion Study in Dutch Health Professionals

**DOI:** 10.3389/fpsyg.2018.01419

**Published:** 2018-09-11

**Authors:** Elena V. Syurina, Zarah M. Bood, Frida V. M. Ryman, Seda Muftugil-Yalcin

**Affiliations:** Athena Institute, Faculty of Science, Vrije Universiteit Amsterdam, Amsterdam, Netherlands

**Keywords:** orthorexia nervosa, western culture, mixed methods research, health professionals, eating disorders, media

## Abstract

Orthorexia Nervosa (ON) is a newly coined eating pattern which disproportionately affects Western countries. Research on the matter is scarce. This study aimed to investigate how the Dutch (mental) health professionals evaluate the influence of “Western culture” on the development of ON. This mixed methods study included interviews (*n* = 15) and a questionnaire (*n* = 157). The extent of influence of the “Western culture” was suggested to be quite high, with a score of 74 out of 100. The factors believed to affect Orthorexia included societal transitions (epidemiological and welfare) and cultural ideas (body ideal and control over life) which, in turn, are influenced by the internet and media. In addition, it was noted that ON is unique among the eating disorders since it does not carry the negative connotations of anorexia or obesity associated with “losing control.” The findings suggest that “Western culture” contributes to the establishment of a high-risk environment for the development of behaviors associated with ON.

## Introduction

Under the global goal of ‘no health without mental health,’ increased attention is being paid to mental health and disorders (Prince et al., 2007). Eating disorders, including anorexia nervosa and bulimia nervosa, are known to constitute a considerable burden within the entirety of mental health problems and have serious consequences on both social and physical health. With mortality rates that are higher than most other mental health problems (Klump et al., 2009), eating disorders in 2013 accounted for 0.1% of global Disability Adjusted Life Years (Erskine et al., 2016).

In recent years, increasing attention has been paid to a new disordered eating pattern, Orthorexia Nervosa (ON) (Dunn and Bratman, 2016). Studies have defined ON as a pathological fixation on eating only foods that are considered to be healthy and pure. Among the beliefs commonly held by the people with this eating pattern are the perceived danger of foods with artificial substances and food prepared with the use of herbicides or pesticides as well as avoidance of certain food groups (i.e., carbohydrates and fats) (Koven and Abry, 2015; Dell’Osso et al., 2016a; Barnes and Caltabiano, 2017). Unlike other eating disorders, such as anorexia nervosa, ON beliefs are focused on the quality of food and not the quantity (Koven and Abry, 2015). However, the danger of ON is not in the beliefs as such, but rather when they lead to the pervasive behaviors that can be detrimental for the quality of life of an individual, such as when maintaining a diet becomes the main priority of the individual and results in neglect in other areas of life (Barnes and Caltabiano, 2017). This can lead to social isolation and deterioration of social contacts of the individual. Additionally, extreme dieting can lead to nutritional deficits or even starvation (Koven and Abry, 2015). Together, the social and biological consequences of such an obsession can substantially reduce the quality of life (Dell’Osso et al., 2016a).

Up until now, research into ON has been scarce, which leads to a number of uncertainties about its etiology, classification, diagnostic criteria, and treatment (Varga et al., 2013). For instance, there is no consensus about whether ON should be defined as a separate disorder (Dunn and Bratman, 2016). Various studies around the world have reported different prevalence ranging from less than 1 to 57.6% (Ramacciotti et al., 2011; Dunn et al., 2017), which can be explained by both sample selection bias and the limitation of the current screening tools (Gleaves et al., 2013). Despite the fact that some studies have investigated the personal predisposition factors, such as being female, university student, underweight, and following a vegetarian or vegan diet (Dell’Osso et al., 2016a, 2018; Hayles et al., 2017; Almeida et al., 2018), research regarding the environmental factors that influence the development of ON is completely lacking. This information would be useful for the facilitation of diagnostic and treatment-oriented research and practice as well as for the general deeper understanding of the phenomenon, its origins, and its prospective development. Prevalence studies conducted till now suggest that ON mostly occurs in Western countries, which leads to the hypothesis of the influence of underlying conditions, such as the culture in these countries (Varga et al., 2013; Dunn and Bratman, 2016). Considering that “Western culture” and its values are being spread around the world due to globalization, an insight into the influence of the “Western culture” on ON is of global importance (Dell’Osso et al., 2016b).

Despite the lack of research into the direct link between ON and “Western culture,” a number of studies have identified it as a direction of interest for further investigation. Some researchers went as far as to suggest that the “Western culture” may be a factor involved in the development of ON (Donini et al., 2004; Varga et al., 2013; Koven and Abry, 2015; Oberle et al., 2017). For instance, Donini et al. (2004) mentioned that the media and the way it creates fear of contaminated food, such as chicken and fish, pressurizes people to pay more attention to healthy and ‘clean’ food. Varga et al. (2013) further stated that the ideals created by the Western society seem to support the ideals that individuals with ON have, i.e., fixation on a healthy lifestyle and achieving slim, muscular bodies. There seems to be a conflict between the societal expectations and the individual possibilities. In order to fit the expectations of the society, people have to make healthy food choices, prepare their own meals, and exercise regularly, which can constitute substantial additional expenses. On the contrary, unhealthy and lower quality food is cheaper and easily accessible. Thus, when an individual is able to maintain a healthy lifestyle, it is praised by the society and viewed as having control over one’s body and life, which is what individuals with ON often strive for. Another factor having an impact on ON could be the increased interconnectedness through social media (Koven and Abry, 2015). In 2017, during an investigation on the link between use of Instagram and ON, it was discovered that a more frequent use of Instagram is significantly linked to a higher risk of developing ON symptoms (Turner and Lefevre, 2017). It is possible that the ability to follow the lives of others, including celebrities, can contribute to the engraining of the beliefs regarding the importance of certain diets.

All these findings combined together support the suggestion that various elements of “Western culture” may be linked to ON just like as it is in the case with anorexia nervosa and bulimia nervosa, which has long been appreciated (Klump et al., 2009). The literature shows that these syndromes are more prevalent in industrialized societies and often “Western cultures” (Pate et al., 1992; Miller and Pumariega, 2001).

Informed by the existing literature, this research strives to make an anthropological investigation into the perceptions of health care professionals in Netherlands regarding the link between “Western culture” and its influence on the development of ON. Having both an emic view on the “Western culture” and also an etic view on the disorder, the health professionals’ ideas can provide valuable contribution to the research question at hand.

The reason to focus on healthcare professionals stems from the fact that the healthcare professionals have an overview of the mental health disorders in general and thus can position ON among other eating disorders and, based on their perspectives, assess its affinity with the “Western culture.” Secondly, healthcare professionals are exposed to ON more than any other group and thus possess rich data and observations regarding ON obtained directly from patients.

## Conceptualization of the “Western Culture”

Before moving on to the description of the methodological set-up of the research, it is important to conceptualize the term “Western culture.” Despite the fact that one can distinguish some trends and beliefs that are common within the “Western culture,” no single definition of such culture exists. This is mostly because culture in itself is a notoriously difficult term to define. One definition of culture that is still widely cited and one that we think is still able to capture its organic nature belongs to Tylor (1958). He states:

“Culture, or civilization, taken in its broad, ethnographic sense, is that complex whole which includes knowledge, belief, art, morals, law, custom, and any other capabilities and habits acquired by man as a member of society.” (Tylor, 1958)

Concurringly, “Western culture” is a term used very broadly to refer to a heritage of social norms, ethical values, traditional customs, belief systems, political systems, and specific artifacts and technologies that have some origin or association with Europe. Among the worldviews that are suggested to be an important part of the “Western culture,” are materialism, individualism, and capitalism (Eckersley, 2006; Hesse-Biber et al., 2006). Materialism, defined as priority given to possessions and money, can be seen as a base for the consumption-based economy in the Western world. Materialistic values tend to reduce the feelings of safety, self-worth, autonomy, and close relationships with others, leading to dissatisfaction among individuals (Eckersley, 2006). Individualism, in which the individual is placed at the center and has freedom of choice, recently got an increasingly prominent role in the “Western culture.” Increased personal expectations, reduced social support, and the search for more self-control, are a result (Eckersley, 2006). Additionally, the capitalistic worldview, in which production is privately owned, creates a strong profit-seeking market within the “Western culture” (Hesse-Biber et al., 2006). Four main industries that were previously linked to eating disorders are distinguished within this market: the food industry, the diet and weight loss industry, the fitness industry, and the cosmetic surgery industry. All the four industries are focused on maximizing profit and respond to the possibilities that the eating disorders create, by drawing upon mass media (Hesse-Biber et al., 2006). Hence, these three worldviews, the industries, and the mass media, which are major elements of the “Western culture,” have all been connected to health risks in the “Western culture” (Eckersley, 2006; Hesse-Biber et al., 2006).

The current study was additionally enriched by the tripartite model by Markey (2004), which presents three pathways for cultural influence on the development of eating disorders. These include transmission of food preferences and restrictions, definition of the body ideals, and perceptions regarding health and disease. These pathways can be transferred to the population level via the use of media, while being influenced by the industry environment and the general cultural views.

## Methodology

This anthropological study was conducted using the mixed methods approach, in particular the concurrent triangulation design. Quantitative data collection was done via an online questionnaire, and qualitative data was collected in the form of semi-structured interviews.

The research was conducted in the Netherlands and no geographical restrictions were imposed. Quantitative data was collected from health professionals throughout the country. For qualitative data collection, the focus areas were Noord-Holland, Zuid-Holland, and Utrecht, because most English-speaking health professionals are located in these areas.

## Sub-Study 1: Questionnaire

### Participants

Professionals, approached via email and social media, included psychologists, psychiatrists, dieticians, and physiotherapists. Only participants with a degree from the university of applied sciences or higher, were eligible to participate. The other inclusion criterion was that the participants needed to be currently clinically active in the Netherlands.

The questionnaire was filled in by 159 participants. However, two participants were removed from the analysis because they did not fulfill the requirements of participation, leaving a total of 157 participants. The majority of these 157 participants was female (87.9%) and the mean age was 44 years. The most frequently occurring profession was dietician with 45.2%, followed by psychologist with 26.1%. Only three psychiatrists participated in answering the questionnaire. Almost 90% of all the participants stated that they had encountered one or more patient(s) with a suspected or confirmed eating disorder in their work. A full overview of the participants’ characteristics can be found in **Table 1**. In order to ensure that all the participants understood ON in a uniform way, a visualization of the current ON criteria was presented prior to the first question (Dunn and Bratman, 2016).

**Table 1:** Characteristics of participants of the questionnaire (*n* = 157).

Variables		Participants (*n* = 157)
Gender^∗^	Male	18 (11.5%)
	Female	138 (87.9%)
	Not listed	1 (0.6%)
	Prefer not to answer	-
Age (in years); mean *(SD)*		43.7 (12.3)
	Missing	7 (4.5%)
Highest level of education^∗^	Specialized pre-university degree	74 (47.1%)
	Bachelor’s degree	10 (6.4%)
	Master’s degree	60 (38.2%)
	Other	12 (7.6%)
	Missing	1 (0.6%)
Profession^∗^	Psychologist	41 (26.1%)
	Psychiatrist	3 (1.9%)
	Dietitian	71 (45.2%)
	Physiotherapist	33 (21.0%)
	Other (namely: GP (*n* = 2), psychotherapist, nutritionist, director of paramedical center, educator in the field of nutrition, psychosocial therapist, and psychosomatic physiotherapist)	8 (5.1%)
	Missing	1 (0.6%)
Years of practice (in years); median (IR)		12.0 (19.0)
	Missing	7 (4.5%)
Language of treatment^∗,∗∗^	English	81 (51.6%)
	Dutch	149 (94.9%)
	Spanish	2 (1.3%)
	French	4 (2.5%)
	Italian	5 (3.2%)
	Other	12 (7.6%)
Patients treated in a language other than Dutch (in %); median (IR)^∗∗∗^		7.0 (9.0)
Encountered patient(s) with a suspected or confirmed eating disorder^∗^	Yes	137 (87.3%)
	No	19 (12.1%)
	Missing	1 (0.6%)


*^∗^Number (%), ^∗∗^multiple answers were possible, ^∗∗∗^this question was only answered by participants who treated patients in both Dutch and one or multiple other language(s).*

### Design

The questionnaire consisted of three parts: diagnosis/classification of ON, influence of “Western culture” on the development of ON, and participants’ characteristics. For the present study, parts two and three of the questionnaire were used, as the results of the diagnosis/classification part of the questionnaire will be reported as a separate study.

The questionnaire has been constructed based on the conceptualization of the “Western culture,” and a tripartite model of pathways linking culture and disordered eating was created for this study. Participants were requested to rate the selected phenomena (see **Appendix A**) using a 5-point Likert scale, for the degree of influence certain phenomena had on the development of ON. The following description of the scales was provided: 1 – no influence at all, 2 – little influence, 3 – moderate influence, 4 – a lot of influence, 5 – great influence.

In his model, Markey has suggested three pathways linking culture and disordered eating, which include eating behaviors, body image ideals, and perceptions of health. For each of the pathways, we have executed a broad internet search to identify the components to be included. The final list of the items was achieved by means of consensus deliberations among participating researchers. The eating behavior factor included two opposing phenomena: availability of fast food and the trend to eat healthy. The body image ideal factors included slim body ideal and muscular body ideal. The perceptions of health factor consisted of attitude toward healthy food (fast-food vs. biological food vs. food low on certain macronutrients) and attitude toward exercise.

Before the questionnaire was distributed, the content and the Dutch-English translation were evaluated four times by different test participants: English and Dutch speaking research master’s students (*n* = 2) and Dutch speaking psychologists (*n* = 2). After each iteration, adjustments were made to the questionnaire based on the oral and written feedback. To test the reliability of the scale, a Cronbach alpha test was conducted. The scale showed an alpha of 0.84. No question, if deleted from the scale, would have increased the alpha. Hence, the scale had a high internal consistency and thus, was reliable.

Furthermore, a principle component analysis was conducted on the 20 items listed in the questionnaire using an orthogonal rotation (varimax). The Kaiser-Meyer-Olkin measure verified the sampling adequacy for the procedure, with KMO = 0.764, “good” according to Hutcheson and Sofroniou (1999). Bartlett’s test of sphericity *χ*^2^(190) = 1028,414, *P* < 0.001, indicated that the correlations between the items were significantly large for the performance of the PCA. Six components had eigenvalues over the Kaiser’s criterion of one and in combination explained 64.5% of variance. After the parallel analysis, only three components were retained. All the factors have a strong reliability measured by Cronbach’s alpha: 0.823, 0.701, and 0.457, respectively. The items included in each component suggest that component 1 represents the possible link between the materialistic and capitalistic beliefs and body ideals, component 2 represents the influence of the food and diet related phenomena while component 3 shows the link between the printed media and the representation of the fashion industry.

### Analysis

Total scores, ranging from 20 to 100, were calculated for each participant in order to investigate the opinions of the participants. A low score means that the participant does not believe that the “Western culture” has an influence on ON, and a high score means that the participant believes that the “Western culture” has a major influence on the development of ON. Results of the questionnaire were analyzed using the software IBM SPSS Statistics 20. The total mean score of mental health and non-mental professionals was calculated separately. An independent samples *t*-test was performed to test whether these total scores differed. Other relations between the total mean score and characteristics of the participants were tested as well, such as gender, age, and experience with eating disorders.

## Results: Questionnaire

### Most Influential Parts of the “Western Culture” on the Development of ON

The mean score was calculated for each individual item of the scale. As shown in **Table 3**, the most influential parts of “Western culture” on ON included digital media (Internet), trends of having healthy foods, and the two perceptions that biological/organic/vegan food and low fat/low carb/gluten free food are the healthiest. The least influential parts of the “Western culture” were considered to be capitalism, materialism, the cosmetic surgery industry, and outdoor advertisements. Overall, the worldviews (individualism, materialism, and capitalism) are seen as the least influential, while the perceptions on what is (un)healthy are seen as the category that has the greatest influence on the development of ON.

### Total Mean Score on the Scale

The total mean score of all the participants (*n* = 150), which is the summed up score of all twenty items on the scale, was a score of 74.0 (out of 100) with a standard deviation of 8.5. The variable total mean score was normally distributed. Only those participants who had filled in all the twenty items of the scale were included in the analysis. The lowest score on the scale was an outlier of 37.0, followed by a lowest score of 54.0, while the highest score was 94.0.

The total mean score (representing the total belief of the link between ON and “Western culture”) was compared among several groups. The total mean score of psychologists and psychiatrists (mental health professionals) was compared to the total mean score of dietitians and physiotherapists (non-mental health professionals). As shown in **Table 2**, the total mean score of the mental health professionals was 72.1, while the total mean score of the non-mental health professionals was 75.0. However, an independent samples *t*-test showed that the difference between the two means was not significant (*P* = 0.063).

**Table 2:** Mean scores, SE, and SD for all factors of the questionnaire.

	Mean	Std. error	Std. deviation
**Worldviews and ideologies**			
Individualism	3.47	0.071	0.883
Materialism	2.71	0.077	0.964
Capitalism	2.60	0.075	0.935
**Market and industries**			
Food industry	3.88	0.073	0.911
Diet and weight loss industry	3.98	0.070	0.873
Fitness industry	3.96	0.070	0.876
Fashion industry	3.64	0.079	0.995
Cosmetic surgery industry	3.11	0.080	1.004
**Mass media**			
Broadcast media (television, movies, and radio)	3.77	0.066	0.831
Digital media (internet)	4.43	0.056	0.700
Printed media (books, newspapers, and magazines)	3.51	0.065	0.807
Outdoor advertisements (billboards, shops, etc.)	3.21	0.073	0.920
**Eating behaviors**			
Availability of fast food	3.41	0.073	0.903
Trends of having healthy diets	4.38	0.050	0.627
**Beauty ideals**			
Thin body ideal	3.81	0.071	0.881
Muscular body ideal	3.71	0.073	0.911
Fast food is unhealthy	4.02	0.068	0.846
**Perceptions of what is healthy**			
Biological/organic/vegan food is the healthiest	4.33	0.057	0.711
Low fat/low carb/gluten-free food is the healthiest	4.26	0.058	0.728
Regular exercise is best for the body	3.78	0.067	0.837


**Table 3:** Total mean score of mental health professionals (*n* = 43) and non-mental health professionals (*n* = 98).

	N (missing)	Mean *(SD)*	Median	Distribution	*T-*value	*P*-value
Psychologists and psychiatrists (mental health professionals)^∗^	43 (1)	72.1 (8.6)	72.0	Normal	-1.874	0.063
Dietitians and physiotherapists (non-mental health professionals)^∗^	98 (6)	75.0 (8.4)	75.5	Normal		


*^∗^The profession category ‘Other’ was not included in this analysis.*

Next, the possible correlation between the age of the respondent and the total score was investigated. Despite the fact that the correlation between the age and score was relatively low (19.1%), a linear regression model showed a significant relationship (*P* = -0.021). In other words, the higher the age of the participants, the more likely they were to have a lower overall score.

Furthermore, the correlation between the total score and gender, highest level of education and years of practice were investigated, but all of them failed to reach significance. Moreover, the linear regression model showed that there was no significant difference in the total score depending on the amount of experience with eating disorders or whether the respondents had first-hand experience with treating an ON patient.

To further investigate the possibility of discrepancy in beliefs between these groups, a non-parametric test, the Mann-Whitney U test, was chosen. A significant difference in the score was found on four items: ‘Digital media’ (*P* = 0.027), ‘Printed media’ (*P* = 0.013), ‘Muscular body ideal’ (*P* = 0.49), and ‘Trends of having healthy diets’ (*P* = 0.037). It appeared that the non-mental health professionals had higher scores on all four items than mental health professionals.

## Sub-Study 2: Interviews

### Participants

The interviews included only mental health professionals, as they are the most likely to treat ON. Participant inclusion was done via email and phone contact with mental health clinics in the selected area. The inclusion criteria for Dutch participants were as follows: should be BIG-registered (official registration stating that the acknowledged education is completed), and should have encountered at least one patient with an eating disorder in the last year. In the case of English-speaking participants the inclusion criteria were as follows: should hold a university degree in psychology/psychiatry, should have a minimum of 3 years clinical experience, and should have encountered at least one patient with an eating disorder in the last year.

A total of fifteen interviews, of which eleven were in Dutch and four were in English (with therapists of non-Dutch origin, namely from Turkey, Norway, Greece, and Romania), were conducted. Thirteen of the fifteen interviews were conducted with psychologists and the other two included psychiatrists. All participants (with the exception of one) were female and the median age of the group was 48 years (age was not normally distributed). On an average, the participants had 16 years of clinical experience. Eleven of the fifteen had considerable experience with treating eating disorders, meaning that they had seen all kinds of eating disorders (i.e., anorexia, bulimia, and Avoidant/Restrictive Food Intake Disorder). Three of them worked in a clinic specializing in eating disorders. Four participants had only seen a few patients with eating disorders. In order to ensure that all the participants understood ON in a uniform way, a visualization of the current characteristics of ON was shown to them prior to the interview.

### Interview Guide

Semi-structured interviews discussed two topics: opinions on diagnosis and classification of ON (including discussion of hypothetical case-study); perceptions about the possible influence of the “Western culture” on the development of ON. This paper will report the outcomes of the second part of the interview alone, as the results of the discussion regarding ON diagnosis are reported elsewhere.

First, the respondents’ demographic data, including profession, age, type of practice, and experience with eating disorders in general and ON in particular, was collected. Next, the participants took part in a mental exercise designed to understand the possible preconceptions and beliefs that the professionals have about individuals with ON-like symptomatology. Respondents were presented with a case represented by an abstract human figure and boxes with the type of symptoms associated with ON (**Appendix B**). The participants were asked about the kind of individual they would envision when looking at this description. They were asked to assign some specific traits such as gender, background, socio-economic position, and personality traits while presenting the patient who was assumed to have ON. After the presentation of the case, the participants were asked what the “Western culture” means to them. Among the probing questions employed during the phase were the ones about the ideologies, industries, and media that are prominent in Europe, North America, and Australia.

Next, the interview led to more in-depth details regarding the influences that the “Western culture” can have on ON. The participants were asked in-depth questions about the reasons for their opinions and how they grew to develop them. The follow-up questions were about the degree to which the participants felt that ON was influenced by “Western culture.” Last, with the help of questions, the participants were provided with opportunities to discuss ON in the context of other cultures.

### Validation Process

Prior to data collection, the interview guide was pilot-tested on three participants (two research master’s students and one psychologist). During each pilot interview, one interviewer conducted the interview and two other researchers were present to evaluate the interview. After the interview, the two researchers and the interviewer discussed the functioning of the interview guide and altered the guide to improve the flow.

### Data Analysis

Interviews were analyzed using ATLAS.ti 7. Thematic analysis was applied to the dataset. Codes were revisited and distributed under the matching themes, creating a coding sheet (**Appendix C**).

### Ethical Considerations

According to the national legislation in the Netherlands (De Wet medisch-wetenschappelijk onderzoek met mensen) and the Institutional guidelines of VU University, no medical ethical approval was required for this study. However, to ensure scientific integrity, written informed consent was obtained from every participant of the study. Additionally, confidentiality of all collected data was guaranteed – all data was stored on a password protected database.

## Results

### Results of the Interviews

#### Mental Visualization of Individuals With ON Symptoms

In addition to the interviews conducted, an additional creative data collection method was deployed at the beginning of each interview. A hypothetical patient with ON was presented to the participants and they were asked to assign the traits to the blank figure representing the patient. Nine out of the fifteen participants expected the person from the infographic to be female; only four said that it could be either female or male. Furthermore, the majority of participants mentioned middle to high education as a characteristic of the hypothetical patient. A notable result is that half of the participants expressed that they saw a Caucasian and/or Western person in front of them: “*I do think it occurs more often in white young girls, or Westernized [girls].*” (Dutch interview 7: Female, 38 years). Asian background was also mentioned, though only twice. However, many respondents also explained that they felt biased by the fact that their patients were mainly Caucasian and Asian individuals.

Related to personality traits, half of the participants agreed that the hypothetical patient was a perfectionist and/or someone with obsessive compulsive traits. Furthermore, rigid, overly ambitious and determined, as well as anxious, were some of the other personality traits that were mentioned.

### Interplay Between the “Western Culture” Phenomena and ON

The aim of the present paper was to investigate the possible link between the “Western culture” and the appearance of ON as a new disordered eating pattern observed throughout the globe. However, during the process of the interview, data collection, and analysis, it became clear that it is impossible to look at the culture in isolation from broader societal changes. In order to present the data in its entirety, a choice was made to start by presenting the underlying societal transitions that form the foundation for occurrence of ON, followed by the discussion of cultural beliefs that can be linked to ON, and finally, a discussion on the means of spreading these beliefs.

### Societal Transitions at Large: Welfare and Epidemiological Transitions

The two major transitions mentioned during the interviews were welfare transition and epidemiological transition. Both of them, apart from being mutually influenced, are closely linked to the changes and developments in the “Western culture” leading to ON.

#### Welfare Transition

The high state of welfare in the “Western culture,” including economic prosperity and well-being of the population, was one of the most prominent themes mentioned by the majority of the participants (ten out of fifteen). An argument to support this theme was that as the economy of the country develops, there is an overall increase in the availability of food and growth in the range of different food types in the market. This growing availability and increasing food security provide a base for more individuals to be able to make nutrition choices. One [ES1] participant said: *“There could be a connection between the nutritional situation and the availability of kinds of food in the Western and the non-Western world. In the West the supply of food may be broader...”* (Dutch interview 1: Female, 31 years).

Another participant drew a link between the changing availability of processed food and an increasing demand for biological products: *“Food has evolved considerably, that it became far away from nature, … that it became more processed and got e-numbers, and you see a reaction on this more and more, like eating original products and non-processed food again.”* (Dutch interview 5: Female, 60 years).

The participants thought that the development and the social economic status of Western countries can lead to the fact that people have less worries about fundamental needs, such as shelter, education, and health care, and thus have time to think about what is healthy, seeing it as a luxurious obsession. In line with the above, the situation was itself directly linked to the pyramid of Maslow by an interviewee. This interviewee said: *“I can imagine that this (Orthorexia) will not occur in the lowest layer [of the Maslow pyramid.]”* (Dutch interview 9: Female, 65 years). Another participant stated: *“you are not going to care about E-numbers when you are hungry, then it would not happen that quickly I think. When a country cannot feed itself, people will not be obsessed with healthy food”* (Dutch interview 3: Female, 51 years).

#### Epidemiological Transitions

The second underlying transition explored during the interviews was the epidemiological transition, which often refers to the changing disease pattern from higher burden of infectious diseases to an increasing burden of non-communicable chronic conditions (Omran, 2005). The role of nutrition and diet is relatively greater for non-communicable conditions, compared to infectious ones, which in turn can contribute to an increasing overall interest in health and healthy food: *“[nowadays] there is a kind of obsession to live a better, purified, to live longer or healthier, …”* (English interview 2: Female, 28 years). The obesity epidemic and the increasing amount of attention given to obesity prevention, contributes to placing healthy food on national and international agendas. It is very interesting to observe that while indeed eating healthy has been accentuated by various public and private actors in western societies, increasing welfare in the same countries paves the way for the production of more processed food than ever. In this awkward moment, the switch from infectious disease to more chronic conditions which are more prone to be influenced by the environment, such as the diet one follows, has the tendency to put the blame on the person who has the disease. This puts pressure on the people and urges them to look for “clean food” in the midst of vast amounts of processed food, simply because they want to fend off possible accusations (or self-accusations) about their own agencies being involved in acquiring the chronic diseases.

### Cultural Influences Informed by the Societal Transitions

The two above described transitions are forming the foundation for the cultural changes that, according to the interviewed health professionals, are believed to have a link to the occurrence of ON. The cultural influences that were mentioned during the interviews, as the ones having the most influence on the development of ON, can be classified under two groups: beliefs linked to the beauty ideals and the ones linked to the lifestyle ideals/control.

#### Beauty Ideals and Weight Loss Industry

Our findings strongly suggested that ON is believed to be connected to the beauty ideals in the “Western culture.” This includes not only striving for thin and muscular bodies but also being healthy and keeping the right image. Even though the main goal of individuals with ON may not always be to lose weight, the interviewees believed that ON is still connected to the thin body ideal as a representation of an ultimately healthy body. The interviewees expressed the opinion that the prevalence of obsession with healthy food can be less in the cultures without the thin body ideal. Furthermore, one of the participants experienced that some of her patients with a history of being overweight were scared that they would become overweight again and hence started to develop ON as a response. On this matter she said: *“I think that that can also have an influence on the development of ON, that you carry on too far, because you are scared of getting overweight again.”* (Dutch interview 7: Female, 38 years).

Several industries were mentioned to have an influence on the development of ON. Among them were the food and the diet/weight loss industries. It was said that these industries were exerting great impact on the society by promoting set beauty ideals and perpetuating the perceived link between a thin body and a healthy body. *“The commercial world is responding to that [beauty ideals] with magazines, but also with people who can make money out of it.”* (Dutch interview 8: Female, 43 years).

#### Lifestyle Ideals and Notion of Control

An increasingly individualistic, perfectionistic, and performance-oriented culture within the Western societies was noted to be a contributing factor for the development of ON, as it also affects how people purchase, consume, and value food. In this regard, one of the interviewees suggested: *“Perhaps it’s also part of the western world, is that you have to think about things. To have an opinion, on a lot of things. And it’s very individualized. … So food is, is a part of it. Because food is, something what you need daily, on a daily basis.”* (English interview 1: Female, 48 years). One of the interviewees went even further by suggesting a possible link between the individualistic nature of the “Western culture” and the increasing prevalence of the mental health problems in general: *“But we are in a very individualistic, the most individualistic, ambitious, … society. So, and, great in many ways, and then also many backsides to that. So that’s why we see all these statistics in, in mental health care and on different conditions that are just racing up for younger generations.”* (English interview 3: Female, 36 years).

According to the participants, one of the major links between the occurrence of ON and the “Western culture” is the commonly held belief about the ideal way of leading one’s life as being in control. Six out of the fifteen participants explained that ON could be a way of seeking shelter and structure in an insecure society. *“Our fundamental needs are met, but our insecurities are emerging more, because religion was gone, having a stable job is not for always, relationships are not for always, more people are divorcing. So people are insecure and are looking for something to fall back on. And then for example Orthorexia or Anorexia is a way to handle insecurities and having comfort like, okay I am doing good in life.”* (Dutch interview 10: Female, 50 years). Such longing for control can be observed in several aspects of life.

Individuals can have the idea that eating healthy will prevent sickness and the look/feel of getting older which implies that you can actually manage your life. *“There is something in Orthorexia that suggests that if we eat and live very healthy, then we do not get sick, we become immortal. So, there is this manufacturability in it, that if you do this, you will never become sick and live forever.”* (Dutch interview 2: Male, 59 years).

Keeping a perfect image or an image which fits the criteria of being perfect according to the prominent beliefs within the society, was suggested to be of importance for ON eating patterns. Talking about people copying what the media told them to eat, an interviewee said: *“In order to consider yourself, worthy enough, or worthy enough to be approved by others, you might adopt these practices [eating healthy and exercising], or similar practices. Because that way, you feel that your self-image gets better, it gets improved somehow.”* (English interview 4: Female, 30 years). Additionally, it was mentioned that nowadays there is often little space for bringing up or discussing negative emotions as people think that this is a sign of “weakness” that one should avoid at all costs. The health professionals discussed a common belief they see in their practice and everyday life, that in order to be successful, an individual should always look happy. On this issue an interviewee said: *“In our culture.. society there is little space for difficult emotions or negative emotions or sad things, resulting in that everybody has to be happy all the time and that is not really feasible. But we harm ourselves a lot because of that.”* (Dutch interview 6: Female, 38 years).

To sum it up, the prominent worldview that suggests that an individual can actually manufacture her own life by being in charge of it, eating healthy, and by actually forcing herself to be happy and strong, is articulated by our interviewees to strongly contribute to the onset of ON.

### Means: Media and Internet

According to all the fifteen interviewees, the media is perceived to play a large role in spreading the cultural views and beliefs that in turn contribute to the development of ON. For instance, when asked about the influence of the “Western culture” on ON, an interviewee stated: *“I think that the media could play a large role in that, with all the fit girl hype kind of scenes. … I also see various societal things happening about hypes that come and go, like the gluten free hype and the vegan hype.”* (Dutch interview 1: Female, 34 years). It was noted that magazines, books, celebrities, advertisements on TV and stores, and especially the Internet, promote healthy food. A participant explained that her patients *“look on Instagram at bloggers, vloggers, etc., Then I get pictures sent to me of the stomach they want to have.”* She continues by saying, *“In that sense, it [Internet] is definitely of influence. Ten/fifteen years ago, this was not an issue.”* (Dutch interview 3: Female, 51 years). Another participant especially underlined the importance of social networks and the bloggers. *“a large part of them [blogs] are of course sponsored, so the industry and the media do have a lot of influence. Too much I think.”* (Dutch interview 3: Female, 51 years).

## Discussion

This mixed methods study aimed to give an overview of the perspectives of the health professionals in Netherlands in relation to “Western culture” and the development of the disordered eating pattern ON. The present paper attempts to conceptualize “Western culture,” dissect it to its various dimensions, and show how each dimension might be linked to the development of ON. Thus, culture is treated as a lens through which meanings are given to processes related to ON in a particular context.

Investigation of the potential link between eating disorders and the cultural environment is not a new phenomenon. The findings linking welfare, media, industries, and beauty ideals, among other things, to development of ON, are consistent with the research connecting the “Western culture” to eating disorders, such as anorexia and bulimia (van den Berg et al., 2002; Hesse-Biber et al., 2006). Additionally, the present study has provided evidence that supports the suggestions previously made by researchers, such as Varga et al. (2013) and Koven and Abry (2015), about the effect of the “Western culture” on an orthorexic eating pattern. However, it is interesting to note that unlike anorexia and bulimia, ON has a different peculiarity which makes it hard to appear as a disorder to an inattentive eye. This is due to the fact that healthy eating and a health conscious life-style are seen as desirable, thus making it difficult to identify when such a positive habit turns into a potential health problem. Our study suggests that while anorexia and bulimia are currently seen as eating disorders that require treatment and are possibly associated with losing control over your food intake, the behavior that is associated with orthorexia is generally seen as being “strong” and “in control” of one’s life. This leads to a suggestion that ON can be potentially seen not as a binary diagnosis, but as a continuum, where the behavior to praise (healthy eating), on one extreme, turns into obsessive traits that can lead to various medical, psychological, and social consequences for an individual with a severe quality of life limitations, on the other extreme. The issue gets more complicated when we understand that it is actually the culture that decides the specific border of acceptance.

Moreover, when discussing such a decisive role of culture in the emergence and progression of eating disorders, it is important to bear in mind that the whole psychopathology of eating disorders is not a static phenomenon, but rather the one that has a tendency to change over time, adapting to the new conditions and environments (Dell’Osso et al., 2016b). Among such continuously evolving and relatively recent factors having a potential influence on the ON is the increasing interconnectivity of the individuals by means of various social media. This idea has previously been reported in the literature by Koven and Abry (2015) and has been noted by the participants of the present study. This has also been reported in the study by Turner and Lefevre, though in their work only one of the social media platforms, namely Instagram, has been associated with ON symptoms. The present study, however, did not limit itself to the investigation of this link alone, but attempted to map broader influences, including the general cultural views, the influence of separate potentially relevant industries, and the impact of mass-media as a means to transfer these views and about the industries. Additionally, we investigated the potential influence of separate beliefs linked to eating behaviors, body image ideals, and perceptions of health. The study partially supports the findings of the predecessors while building on them and creating a base for further, and more in-depth investigation on the matter.

The overall findings showed that while the vast majority of participants (both in qualitative and quantitative sub-studies) seemed to believe that such a relation existed, the group differences reflect the group variations. Despite the fact that the group differences between mental health and non-mental health professionals regarding the overall total score on the survey failed to reach significance, the fact that some inter-group variation in opinion has been observed opens a new line of inquiry. For instance, non-mental health professionals found variables, such as Digital media, Printed media, Muscular body ideal, and Trends of having healthy diets to be significantly more influential for the development of ON.

We have to note that the health professionals, who were the focus of the present study, are also part of the culture that they operate in and this should be taken into consideration when discussing both the outcomes of the study and the medical-oriented research on the matter. We believe that in order to expand on the presented findings, a more deeper research concerning the separate parts of the “Western culture” and their possible link to ON should be carried out to investigate the precise mechanisms behind them. In addition, semi-experimental studies comparing the relation between ON and various degrees of exposure to health-promoting media could provide an insight into the effect of such media on the development of ON. Another important source of the data could be a study investigating the beliefs of the non-health-related individuals, potentially those who self-identify as having ON-related problems. Additionally, relatives or caretakers close to the experiential expert could also provide useful information regarding the influence of media and other influential factors, because they are close to the process of developing a pathological eating pattern.

To be able to provide empirical evidence of the link between the “Western culture” and ON, adequate prevalence studies should test the hypothesis that ON is more prevalent in areas with the “Western culture.” However, it should be noted that “Western culture” cannot be geographically defined. Even within the ‘Western world’ (i.e., North-America, Europe, etc.), there are major differences in culture between the countries and within the countries. In the present study it was therefore hard to prevent the participants from answering questions with their own perception of the “Western culture” in mind. In order to minimize this in interviews, the interview began with a discussion on what the “Western culture” entails. Prevalence studies should take into account the fact that the boundaries of “Western culture” are indistinct. Thus while measuring prevalence, one could for instance measure and compare the prevalence of ON in main cities of Western countries, as well as in more rural areas.

Insight into how the “Western culture” affects ON is not only valuable for a more anthropological and aetiological discussion about ON, but can be valuable for possible future interventions and treatment. When the possible links are identified, the interventions could focus on tackling some of these risk factors. Examples include supervising what is promoted by media and providing more information about the scientific background of diets. Treatment of ON would rather take these factors into account, while focusing on changing the eating patterns and cognitions of the patient. For instance, when treating ON, the health professionals should keep in mind that their patients are greatly affected by the media and society.

Reflecting on the methods used in the study, some limitations should be mentioned. For instance, the number of respondents on the questionnaire was not equally distributed among the four professions. When comparing mental health professionals with non-mental health professionals, the mental health professionals were greatly outnumbered, contributing to only 28% of the total responses against 66% by non-mental health professionals. In further research, the representation of the professions could be made more balanced in order to prevent bias. Next, the number of interviews that were conducted was limited. However, the fact that the data saturation was achieved in the present study suggests that no other findings would have been reported if a greater number of interviews was conducted.

In sum, the “Western culture” is believed to have a considerable influence on the development of ON. One of the most frequently mentioned reasons for this is that the high state of welfare within the culture enables people to choose healthy food, while on the other hand promoting the prevention of obesity. Furthermore, the media and industries, such as the food industry, the diet and weight loss industry, and the fitness industry, have great power in creating an obsession with health, healthy food, and thin beauty ideals among the citizens in the “Western culture.” The individualistic and perfectionistic character of the “Western culture” adds to this by creating insecurity and a need for control.

## Author Contributions

All authors participated in conceptualization of the research. ZB and FR collected the data under the supervision of ES. Primary analysis was done by ZB, FR, and ES contributed to in depth analysis and the final conceptualization of the work. ES wrote the article with continuous feedback from the other co-authors. SM-Y provided assistance in conceptualization of the cultural background.

## Conflict of Interest Statement

The authors declare that the research was conducted in the absence of any commercial or financial relationships that could be construed as a potential conflict of interest.

## Figures and Tables

**FIGURE A1:**
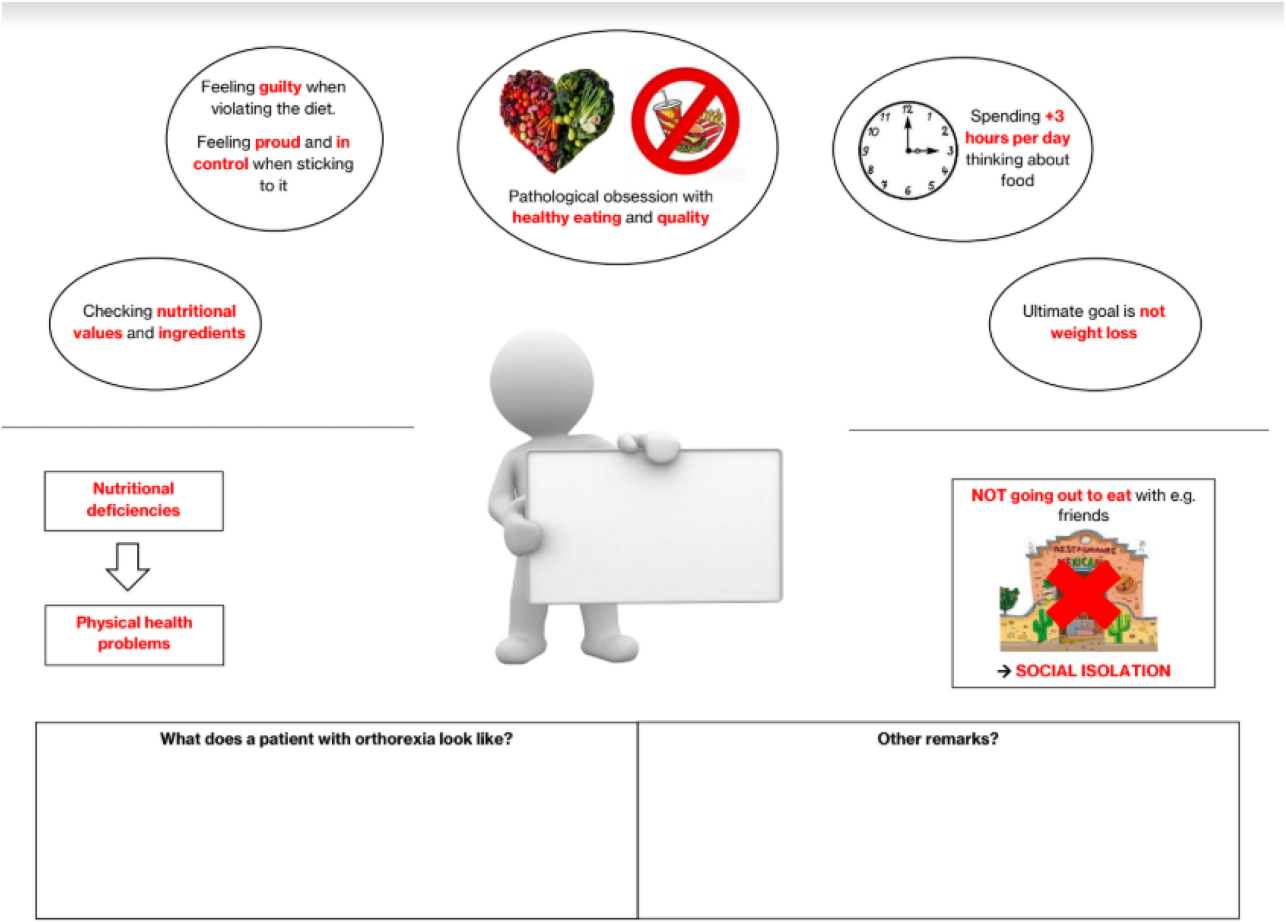
Schematic representation of an individual with ON-related symptoms used during interviews.

**Table:** 

***1. How is the development of ON influenced by the modern Western culture?***	
1.1. Welfare	1.1.1. Availability of food
1.1.2. Option to only eat healthy	
1.1.3. Development and SES of country	
1.2. Processes within society	1.2.1. Obsession with health and healthy food
1.2.2. Attention for food safety	
1.2.3. Ignorance to non-scientific background	
1.2.4. Food has become a religion or culture	
1.2.5. Respond on food being far away from nature	
1.2.6. Way of seeking control	
1.2.7. Reaction on obesity epidemic and obesity prevention	
1.3. Worldviews and ideologies	1.3.1. Perfectionism and performance-orientated
1.3.2. Individualism	
1.3.3. Capitalism	
1.4. Media	1.4.1. Food hypes
1.4.2. Much attention to healthy food	
1.4.3. Printed media	
1.4.4. Internet	
1.4.5. Celebrities	
1.4.6. Too much input	
1.4.7. Information spreads more quickly	
1.5. Industries	1.5.1. Food industry
1.5.2. Diet and weight loss industry	
1.5.3. Have a lot of power	
1.6. Manufacturability of life	1.6.1. Idea that eating healthy prevents sickness and getting old
1.6.2. Eating healthy makes you closer to perfect	
1.6.3. You need to be happy; no place for negative	


## Appendix A. Questionnaire Structure

### Introduction

This questionnaire is part of a research project about Orthorexia Nervosa. Orthorexia Nervosa is an emerging disordered eating pattern, in which the individual acquires a pathological obsession with healthy eating. This can have physical, psychological and social consequences. As it is a relatively new condition, little research has been conducted on it. There is no consensus regarding diagnostic criteria, and the studies that have been conducted on it are inconclusive.

The aim of this study is to shed light on the perspectives of health professionals on the condition, current diagnostic criteria, possible influential factors and how Orthorexia Nervosa should be classified. The required data is collected through interviews and this questionnaire.

The questionnaire will consist of three chapters:

- Chapter 1: Recognition, diagnosis, and classification of Orthorexia.

- Chapter 2: Influence modern Western culture on Orthorexia.

- Chapter 3: Characteristics of respondent.

Overall, it will take approximately 10 min to fill in the questionnaire. All your answers will stay anonymous. If you would like to receive the results of the research, you can send an email to orthorexiaresearch@gmail.com. In this way, your email cannot be linked to your response on the questionnaire.

### Chapter 1: Recognition, Diagnosis, and Classification of Orthorexia

#### Recognition and Diagnosis

This chapter consists of questions regarding recognition, diagnosis and classification of Orthorexia Nervosa.

- > Picture of criteria of ON here

Have you met clients who fulfill these criteria? 1. Yes, within the last year

2. Yes, more than 1 year ago
3. No

How prevalent do you the think the condition is in the general population in the Netherlands (1 meaning not at all prevalent and 5 meaning extremely prevalent)?

1 2 3 4 5

Do you think Orthorexia Nervosa should have its own diagnosis in the upcoming versions of the DSM?

Yes/No

*If no*, which existing diagnosis do you think it fits within? *Multiple options are possible*

1. Anorexia Nervosa
2. Bulimia Nervosa
3. Avoidant/Restrictive Food Intake Disorder (ARFID)
4. Obsessive and Compulsive Disorder
5. General Anxiety Disorder
6. Other:_____________

#### Classification

In the DSM-5, diagnoses are divided into different categories of diseases or disorders of the same type.

Which diagnostic category or categories does Orthorexia Nervosa fall under according to your opinion?

Multiple options are possible

1. Eating and Feeding Disorders
2. Obsessive Compulsive Disorders
3. Anxiety Disorders
4. Other: ______________

#### Additional Factors

In the media, several factors are presented as possible contributors or part of the condition.

On a scale from 1 to 5, to what extent do you think each of the following factors contribute to/are part of Orthorexia Nervosa (1 meaning not a contributor/not part of the condition at all and 5 meaning a vital contributor/great part of the condition)?

• Exercise 1 2 3 4 5
• Weight loss 1 2 3 4 5

Do you think exercise related symptoms should be part of the diagnostic criteria?

Yes/No

### Chapter 2: Influence Modern Western Culture on Orthorexia

In this chapter you will get questions regarding a possible influence of the modern Western culture on the emergence of Orthorexia. This questionnaire will not ask about other factors that could influence orthorexia, but the fact that they are prevalent is acknowledged.

On scale of 1–5, with 1 meaning no influence at all and 5 meaning a great influence, to what extent do you consider each of the following parts of the modern Western culture to influence the emergence of Orthorexia?

Worldviews and values in the modern Western culture:

• Individualism 1 2 3 4 5
• Materialism 1 2 3 4 5
• Capitalism 1 2 3 4 5

Profit-seeking market and its industries:

• Food industry 1 2 3 4 5
• Diet- and weight loss industry 1 2 3 4 5
• Fitness industry 1 2 3 4 5
• Fashion industry 1 2 3 4 5
• Cosmetic surgery industry 1 2 3 4 5

Mass media:

• Broadcast media (Television, movies, and radio) 1 2 3 4 5
• Digital media (Internet) 1 2 3 4 5
• Printed media (Books, newspapers, and magazines) 1 2 3 4 5
• Outdoor advertisements (Billboards, shops, etc.) 1 2 3 4 5

The worldviews, values, industries, and the mass media prevalent in the modern Western culture, shape perceptions and behaviors of individuals. On scale of 1–5, with 1 meaning no influence at all and 5 meaning a great influence, to what extent do you consider the following perceptions and behaviors within the modern Western culture to influence the emergence of Orthorexia?

Beauty ideals within the modern Western culture:

• Thin body ideal 1 2 3 4 5
• Muscular body ideal 1 2 3 4 5

Perceptions within the modern Western culture on what is (un)healthy:

• Fast food is unhealthy 1 2 3 4 5
• Biological/organic/vegan food is the healthiest 1 2 3 4 5
• Low fat/low carb/gluten free food is the healthiest 1 2 3 4 5
• Regular exercise is best for the body 1 2 3 4 5

Eating behaviors within the modern Western culture:

• Eating fast food 1 2 3 4 5
• Trends of having healthy diets (organic, low carb, etc.) 1 2 3 4 5

### Chapter 3: Characteristics of Respondent

This chapter consists of questions regarding your characteristics.

Gender 1. Male

2. Female
3. Not listed: ________
4. Prefer not to answer

Age _______________________

Ethnicity 1. Caucasian

2. Black or African American
3. Asian
4. Native Hawaiian or Pacific Islander
5. Other: ___________

Highest level of education 1. HBO

2. Bachelor’s Degree
3. Master’s Degree
4. Other: ___________

What is your profession? 1. Psychologist

2. Psychiatrist
3. Dietitian
4. Physiotherapist

For how many years have you been practicing this profession? _______________________

What languages do you treat patients in?

Multiple options possible.

1. Dutch
2. English
3. Spanish
4. French
5. Italian
6. Other: ____________

Have you encountered patient(s) with a suspected or confirmed eating disorder in the clinic? Yes/No

End

Do you have anything you would like to add or comment on?

_______________________________________________________________________

_______________________________________________________________________

_______________________________________________________________________

_______________________________________________________________________

Do you have any advice or comments regarding the questionnaire itself?

_______________________________________________________________________

_______________________________________________________________________

_______________________________________________________________________

_______________________________________________________________________

If you would like to receive the results of this study or if you have any questions, you can send an email to *orthorexiaresearch@gmail.com*.

Thank you for your participation!

## Appendix B. Interview Guide

### Introduction

Welcome (name participant),

First of all we would like to thank you for your participation in this study and for allowing us to have this interview with you. My name is *(Alice/Frida)*, and I am conducting this interview as part of a study about professional’s opinions regarding a newly emerging eating pattern called orthorexia nervosa. The other researchers involved in this study are *(Alice/Frida/Zoë)*. We will be conducting this study as a part of the research Master’s in Global Health at the Vrije Universiteit Amsterdam and the Bachelor program European Public Health at Maastricht University. The principal researcher of this study is Elena Syurina. She is an assistant professor at the Vrije Universiteit Amsterdam as well as at Maastricht University and she will be guiding this study.

The objective of the research project is to gain insight into the perspectives of health professionals in the Netherlands regarding Orthorexia Nervosa. Individuals with Orthorexia Nervosa acquire a pathological obsession with healthy eating. Little research has been conducted into the condition. Therefore, there is no consensus regarding diagnostic criteria, and the studies that have been conducted on it are inconclusive. By means of interviews and a questionnaire this study will explore how health professionals in the Netherlands view the condition, its diagnostic criteria, factors that influence it and how it should be classified. This will result in improved knowledge and understanding of the condition and facilitate the development of treatments for it, leading to better clinical care for patients.

As for this interview, feel free to ask questions whenever you want, and take as much time as you need to answer the questions. You are not obligated to answer a question if you do not feel comfortable to do so. Participation is voluntary and you may withdraw from the study at any given time without giving reasons. Please note that we handle the information provided to us with the utmost care. All your data will be anonymized and stored at a safe and password protected database. All personally identifiable information will be deleted during the analysis of the data, so that no one can trace your answers back to you. Also, during the interview I will provide you with an infographic with information about a fictional patient, with which I would like to work interactively with you. You will be provided with more information about this later in the interview. Finally, I would like to ask for your permission to record this interview and to analyze the answers provided for the purpose of this study.

Now that you know more about the research, are you still willing to participate in the study? **[if yes]** Would you be so kind to sign the informed consent form?

**[PROVIDE PARTICIPANT WITH INFORMED CONSENT FORM]**

If it is alright with you, I would like to turn on the recorder now?

**[TURN RECORDER ON IF ALLOWED, OTHERWISE MAKE SURE THAT YOU HAVE YOUR PENS AND PAPER READY AND AT HAND]**

Now, on record, is it okay if we record and use the data from this interview for the purposes of this project?

### Background Information Interviewee

I would like to start off by asking you some questions about yourself. This data will be used as background information during the analysis of the results.

• How old are you?
• Where are you from?
• For how long have you been working as a *psychologist/psychiatrist*?
• Where did you go to school? What type of study program did you follow? Did you take any additional courses/certificates that could be of interest for this study [*e.g., a course about eating disorders]*?
• Type of practice that you are working in?/What type of patient group *[e.g., children, veterans, immigrants, only OCD patients and inpatient or outpatient care]*
• Do you also treat patients in Dutch? Could you estimate the proportion of people whom you treat in English or another foreign language, versus the proportion of people whom you treat in Dutch?
• What experience do you have with patients with eating disorders?
• What types of eating disorders did you encounter during your work as a *psychologist/psychiatrist? [e.g., anorexia, bulimia, binge eating disorder].*
• How many patients (approximately) did you treat for having an eating disorder during the past year/month/week?
• What other types of conditions do you see in your work?
• OCD, personality disorders, and anxiety disorders?
• How many patients (approximately) did you treat for having OCD/personality disorders/anxiety disorders during the past year/month/week?

### Diagnosis and Classification

I will now show you a picture of a hypothetical patient who might come into your clinic and I will ask you some questions about this patient.

**[NOTE: provide participant with the picture and some whiteboard markers: walk the participant through the picture so that you are sure that he/she understands it completely, also explain that he/she can fill in the possible diagnosis in the box in the center].**

Let us say that a patient presents with these symptoms.

• Who do you see in front of you? Do you imagine this to be a specific patient? How would you describe the characteristics/personality of this patient? *There are no right or wrong answers, I would just like to know what you think.* **[NOTE: ask them to write/draw on the picture]**
• (Age, gender, ethnicity, education etc)
• Are you able to diagnose this person based on current classifications, such as the DSM 5?
• ***If yes*** → How would you diagnose this patient?
• Why do you think it should be diagnosed like this? Why shouldn’t it be diagnosed as something else? (e.g., when they state that it is bulimia, why isn’t it anorexia?)
• Is the diagnosis sufficient? If yes, why and if no, why not?
• Do you see these kind of patients in your work? How often?
• ***If no*** → Why not? What else would you need to know?

Currently the proposed diagnostic criteria of Orthorexia Nervosa, which is the condition that we are investigating, are these **[NOTE: show a table with the criteria from Dunn and Bratman. Walk them through the criteria].** Could you please have a look at it?

• So now that you have been presented with another possible diagnosis, would you change the diagnosis of the hypothetical patient? Or do you still feel that the diagnosis you were able to give according to current classifications is more fitting? Why?
• ***If the original diagnosis fits better*** → does that mean that you do not think it should have its own classification?
• Why/why not?
• Advantages/disadvantages?
• Do you think that a separate diagnosis for ON would contribute to improved treatment possibilities for patients? Do you think that this is important? Why/why not? [*probably depends on the number of orthorexic patients that they see]*
• Do you see patients who you believe fit the criteria for orthorexia nervosa? (*if yes*, ask them how often they see them)
• ***If the orthorexia diagnosis fits better*** → does that mean that you think it should have its own classification?
• Why/why not?
• Advantages/disadvantages?
• Do you think that a separate diagnosis for ON would contribute to improved treatment possibilities for patients? Do you think that this is important? Why/why not? [*probably depends on the number of orthorexic patients that they see]*

**[NOTE: show them the list of possible diagnostic categories in the DSM V]**

Looking at the current diagnostic categories in the DSM 5, where do you think orthorexia fits in? To which category or categories does it belong? **(NOTE: there are multiple possibilities and no wrong answers)**

• **If more than one category is mentioned** →
• Could you explain why you think it fits in multiple categories?
• If you had to choose one, where do you think it would fit best?
• Do you see patients who you believe could fit the criteria for orthorexia nervosa?(if yes, ask them how often they see them)

### Influence Modern Western Culture

Now I would like to talk to you about another aspect of orthorexia, namely the link between orthorexia and culture. Orthorexia is mainly found to be prevalent in developed countries, meaning that there COULD be a link between the Western culture and the emergence of orthorexia. However, no research has yet been conducted on this. I would like to discuss the possibility of this link with you.

• First I would like to know, what do you perceive as part of the modern Western culture?

**(NOTE: If the interviewee is stuck or does not have a sufficient idea about it, you can help them with this: *Modern Western culture (in Europe, North America, Australia) consists of:***

***Worldviews and values like individualism, materialism, and capitalism***

***Profit seeking industries; food, diet- and weightloss, and fitness industries***

***Mass media (used by the industries))***

• What are your thoughts on the link between the modern Western culture and orthorexia?
• Do you think that the modern Western culture influences the emergence of orthorexia?

***If suggested that there is a link:***

• Why do you think that?
• How does the modern Western Culture influence the emergence of orthorexia? In what ways? (**NOTE: You can think about influencing eating behaviors, body image ideals, and perceptions of health/illness. But do not provide this to them unless they really have nothing to work with.)**
• To what degree does the modern Western culture influence the emergence of ON? Is there a strong or minor influence?
• What about other cultures? Will other cultures be linked to ON or it is only the Western culture?

***If not suggested that there is a link:***

• Why not? Can you elaborate?
• What about other cultures? Will other cultures be linked to ON?

End

That was the last question I had for now, is there anything else you would like to add?

To finish off, I would, again, like to ask you if it is okay with you that the data from this interview is used within the study.

Finally I would like to thank you for taking the time to do this interview. If you would like, I can send you the end-product (report, article) when it is finished. You can also get a summary or transcript of the interview if you want.

## References

[B1] Almeida C. Borba V. V. Santos L. (2018). Orthorexia nervosa in a sample of portuguese fitness participants. *Eat. Weight Disord.* 23 443–451. 10.1007/s40519-018-0517-y 29808255

[B2] Barnes M. A. Caltabiano M. L. (2017). The interrelationship between orthorexia nervosa, perfectionism, body image and attachment style. *Eat. Weight Disord.* 22 177–184. 10.1007/s40519-016-0280-x 27068175

[B3] Dell’Osso L. Abelli M. Carpita B. Massimetti G. Pini S. Rivetti L. (2016a). Orthorexia nervosa in a sample of Italian university population. *Riv. Psichiatr.* 51 190–196. 10.1708/2476.25888 27869905

[B4] Dell’Osso L. Abelli M. Carpita B. Pini S. Castellini G. Carmassi C. (2016b). Historical evolution of the concept of anorexia nervosa and relationships with orthorexia nervosa, autism, and obsessive-compulsive spectrum. *Neuropsychiatr. Dis. Treat.* 12 1651–1660. 10.2147/ndt.s108912 27462158 PMC4939998

[B5] Dell’Osso L. Carpita B. Muti D. Cremone I. Massimetti G. Diadema E. (2018). Prevalence and characteristics of orthorexia nervosa in a sample of university students in Italy. *Eat. Weight Disord.* 23 55–65. 10.1007/s40519-017-0460-3 29134507

[B6] Donini L. M. Marsili D. Graziani M. P. Imbriale M. Cannella C. (2004). Orthorexia nervosa: a preliminary study with a proposal for diagnosis and an attempt to measure the dimension of the phenomenon. *Eat. Weight Disord.* 9 151–157. 10.1007/BF03325060 15330084

[B7] Dunn T. M. Bratman S. (2016). On orthorexia nervosa: a review of the literature and proposed diagnostic criteria. *Eat. Behav.* 21 11–17. 10.1016/j.eatbeh.2015.12.006 26724459

[B8] Dunn T. M. Gibbs J. Whitney N. Starosta A. (2017). Prevalence of orthorexia nervosa is less than 1%: data from a US sample. *Eat. Weight Disord.* 22 185–192. 10.1007/s40519-016-0258-8 26902744

[B9] Eckersley R. (2006). Is modern Western culture a health hazard? *Int. J. Epidemiol.* 35 252–258. 10.1093/ije/dyi235 16303804

[B10] Erskine H. E. Whiteford H. A. Pike K. M. (2016). The global burden of eating disorders. *Curr. Opin. Psychiatry* 29 346–353. 10.1097/yco.0000000000000276 27532942

[B11] Gleaves D. H. Graham E. C. Ambwani S. (2013). Measuring’orthorexia’: development of the eating habits questionnaire. *Int. J. Educ. Psychol. Assess.* 12 1–18.

[B12] Hayles O. Wu M. S. De Nadai A. S. Storch E. A. (2017). Orthorexia nervosa: an examination of the prevalence, correlates, and associated impairment in a university sample. *J. Cogn. Psychother.* 31 124–135. 10.1891/0889-8391.31.2.124 32755933

[B13] Hesse-Biber S. Leavy P. Quinn C. E. Zoino J. (2006). “The mass marketing of disordered eating and eating disorders: the social psychology of women, thinness and culture. *Paper Presented at the Women’s Studies International Forum*, New York, NY, 208–224. 10.1016/j.wsif.2006.03.007

[B14] Hutcheson G. D. Sofroniou N. (1999). *The Multivariate Social Scientist: Introductory Statistics Using Generalized Linear Models*. Thousand Oaks, CA: Sage 10.4135/9780857028075

[B15] Klump K. L. Bulik C. M. Kaye W. H. Treasure J. Tyson E. (2009). Academy for eating disorders position paper: eating disorders are serious mental illnesses. *Int. J. Eat. Disord.* 42 97–103. 10.1002/eat.20589 18951455

[B16] Koven N. S. Abry A. W. (2015). The clinical basis of orthorexia nervosa: emerging perspectives. *Neuropsychiatr. Dis. Treat* 11 385–394. 10.2147/ndt.s61665 25733839 PMC4340368

[B17] Markey C. N. (2004). Culture and the development of eating disorders: a tripartite model. *Eat. Disord.* 12 139–156. 10.1080/10640260490445041 16864313

[B18] Miller M. N. Pumariega A. J. (2001). Culture and eating disorders: a historical and cross-cultural review. *Psychiatry* 64 93–110. 10.1521/psyc.64.2.93.18621 11495364

[B19] Oberle C. D. Samaghabadi R. O. Hughes E. M. (2017). Orthorexia nervosa: assessment and correlates with gender. BMI, and personality. *Appetite* 108 303–310. 10.1016/j.appet.2016.10.021 27756637

[B20] Omran A. R. (2005). The epidemiologic transition: a theory of the epidemiology of population change. *Milbank Q.* 83 731–757. 10.1111/j.1468-0009.2005.00398.x 16279965 PMC2690264

[B21] Pate J. E. Pumariega A. J. Hester C. Garner D. M. (1992). Cross-cultural patterns in eating disorders: a review. *J. Am. Acad. Child Adolesc. Psychiatry* 31 802–809. 10.1097/00004583-199209000-00005 1345557

[B22] Prince M. Patel V. Saxena S. Maj M. Maselko J. Phillips M. R. (2007). No health without mental health. *Lancet* 370 859–877. 10.1016/s0140-6736(07)61238-0 17804063

[B23] Ramacciotti C. Perrone P. Coli E. Burgalassi A. Conversano C. Massimetti G. (2011). Orthorexia nervosa in the general population: a preliminary screening using a self-administered questionnaire (ORTO-15). *Eat. Weight Disord.* 16 e127–e130. 10.1007/BF03325318 21989097

[B24] Turner P. G. Lefevre C. E. (2017). Instagram use is linked to increased symptoms of orthorexia nervosa. *Eat. Weight Disord.* 22 277–284. 10.1007/s40519-017-0364-2 28251592 PMC5440477

[B25] Tylor E. B. (1958). *Primitive Culture* (1871). New York, NY: JP Putnam’s Sons.

[B26] van den Berg P. Thompson J. K. Obremski-Brandon K. Coovert M. (2002). The Tripartite Influence model of body image and eating disturbance. *J. Psychosom. Res.* 53 1007–1020. 10.1016/S0022-3999(02)00499-3 12445590

[B27] Varga M. Dukay-Szabo S. Tury F. van Furth E. F. (2013). Evidence and gaps in the literature on orthorexia nervosa. *Eat. Weight Disord.* 18 103–111. 10.1007/s40519-013-0026-y 23760837

